# Plasma CHI3L1 in Amyotrophic Lateral Sclerosis: A Potential Differential Diagnostic Biomarker

**DOI:** 10.3390/jcm12062367

**Published:** 2023-03-19

**Authors:** Alessandro Bombaci, Umberto Manera, Giovanni De Marco, Federico Casale, Paolina Salamone, Giuseppe Fuda, Giulia Marchese, Barbara Iazzolino, Laura Peotta, Cristina Moglia, Andrea Calvo, Adriano Chiò

**Affiliations:** “Rita Levi Montalcini” Department of Neuroscience, University of Turin, 10126 Turin, Italy

**Keywords:** biomarker, chitinases, cognitive impairment, differential diagnosis, early diagnosis, MND mimics, red blood cells

## Abstract

(1) Background: Motor neuron diseases (MNDs) are fatal neurodegenerative diseases. Biomarkers could help with defining patients’ prognoses and stratifications. Besides neurofilaments, chitinases are a promising family of possible biomarkers which correlate with neuroinflammatory status. We evaluated the plasmatic levels of CHI3L1 in MNDs, MND mimics, and healthy controls (HCs). (2) Methods: We used a sandwich ELISA to quantify the CHI3L1 in plasma samples from 44 MND patients, 7 hereditary spastic paraplegia (HSP) patients, 9 MND mimics, and 19 HCs. We also collected a ALSFRSr scale, MRC scale, spirometry, mutational status, progression rate (PR), blood sampling, and neuropsychological evaluation. (3) Results: The plasma levels of the CHI3L1 were different among groups (*p* = 0.005). Particularly, the MND mimics showed higher CHI3L1 levels compared with the MND patients and HCs. The CHI3L1 levels did not differ among PMA, PLS, and ALS, and we did not find a correlation among the CHI3L1 levels and clinical scores, spirometry parameters, PR, and neuropsychological features. Of note, the red blood cell count and haemoglobin was correlated with the CHI3L1 levels (respectively, *p* < 0.001, r = 0.63; *p* = 0.022, and r = 0.52). (4) Conclusions: The CHI3L1 plasma levels were increased in the MND mimics cohort compared with MNDs group. The increase of CHI3L1 in neuroinflammatory processes could explain our findings. We confirmed that the CHI3L1 plasma levels did not allow for differentiation between ALS and HCs, nor were they correlated with neuropsychological impairment.

## 1. Introduction

Motor neuron diseases (MNDs) are fatal neurodegenerative diseases that are mainly characterized by a progressive death of motor neurons. Their pathological mechanisms are complex and heterogeneous [1]. The most common form of MND is amyotrophic lateral sclerosis (ALS), which is characterized by a progressive loss of upper and lower motor neurons, which causes weakness of the bulbar, limb, trunk, and respiratory muscles [2]. Around 10% of ALS cases are familial (fALS), whilst 90% are sporadic (sALS) [3], and up to 50% of ALS patients have a cognitive and/or behavioral impairment [1]. The aetiology and pathophysiology of ALS are still unknown, and its prognosis generally occurs approximately three years after its diagnosis. Other forms of MNDs include primary lateral sclerosis (PLS), which is characterized by exclusive damage to the upper motor neurons, and progressive muscular atrophy (PMA), a form that involves primary lower motor neuron damage.

There is a strong need to identify the prognostic and diagnostic biomarkers that allow for an early differentiation of ALS from ALS mimics, patients’ stratification, and a prediction of the disease outcome, in order to improve patients’ inclusion in clinical trials. Neurofilaments (NFs) are now becoming a widely accepted prognostic biomarker for ALS and other neurodegenerative diseases [4,5].

More recently, chitinases, a family of hydrolytic enzymes, have been studied in the cerebrospinal fluid (CSF) of ALS patients as possible liquid biomarkers. They include CHIT1, CHI3L1, and CHI3L2, and correlate with neuroinflammatory status [6,7]. CHIT has been extensively studied in the CSF of ALS patients [6,7,8,9,10] and investigated in plasma [6], showing a promising role in differential diagnosis. Conversely, CHI3L1 has been less studied. Moreover, CHI3L1 is also involved in processes of systemic inflammation, such as autoimmune diseases [11], cancers [12], liver diseases [13], and diabetes [14]. A recent study showed that CHI3L1 is highly expressed in the activated astrocyte in the motor cortex of ALS patients [6]. Additionally, Thompson and colleagues observed that the CSF levels of CHI3L1 correlate with cognitive impairment in ALS [7]. This is an interesting result, since a validated biomarker that is related to cognitive impairment does not exist, and needs to be confirmed in plasma. Based on these premises, we decided to carry out a pilot study in order to compare the levels of the CHI3L1 in the plasma samples gathered during the first visits to our center, which were obtained from MND patients, MND mimics, and healthy controls (HCs). We decided to focus on plasma, because blood sampling is an easier and less invasive procedure compared to cerebrospinal puncture.

## 2. Materials and Methods

### 2.1. Clinical Assessment

The MND patients received their diagnosis following the Gold Coast criteria [15] and the PLS criteria [16]. Their disease severity was assessed using the amyotrophic lateral sclerosis revised Functional Rating Scale (ALSFRSr) [17]. We excluded patients with active cancer, infectious diseases, autoimmune diseases, diabetes, and liver diseases, and those ongoing immunosuppressive therapies.

All the patients underwent blood sampling in their first visit to our ALS Centre.

Moreover, all the MND patients underwent a clinical evaluation (a body mass index [BMI] measurement, neurological examination, including a Medical Research Council scale [MRC] [18] measurement, and the ALSFRSr scale), general blood tests, an instrumental evaluation (electromyography, a cerebral MRI with tractography, and functional MRI, spirometry), a neuropsychological evaluation [19] (including MMSE, ECAS, TMT-A, TMT-B, RAVLT, ROCF, FAB, Digit Span, and FRSBE), and a genetic test for the most common mutations (*C9orf72, SOD1*, *TARDBP,* and *FUS* genes). The patients’ phenotypes were classified as follows: classic ALS, bulbar ALS, flail arm, flail leg, and prevalent upper motor neuron ALS [2].

The progression rate (PR) was calculated as the (48—ALSFRSr at blood sampling)/time interval of the months between the symptoms onset and blood sampling. Fast- and slow-progression ALS were divided using the median values of the PR (0.54 points/month).

### 2.2. Laboratory Evaluation

From each patient and control, we collected 4 mL of fresh blood in an EDTA tube. The blood samples were centrifuged at 20 °C at 1500× *g* for 15 min within 1 h from collection, and the obtained plasma was stored at −80 °C.

The samples were kept at 4 °C overnight before the ELISA test to allow for slow thawing; then they were left at room temperature for 1 h and used to run the test.

We used the Human Chitinase-3-Like Protein 1 (CHI3L1) ELISA commercial Kit (Abbexa, Cambridge, UK), following the manufacturer’s instructions. All the ELISA assays were performed in duplicate. The plates were read using a CLARIOstar Plus plate reader (BMG LABTECH, Ortenberg, Germany). The standard curves were fitted with 4-parameter logistic regression, using MARS data analysis software (BMG LABTECH). The samples were diluted 1:40 in order to achieve a concentration within the linear range of the standard curve measurements. The median intra-assay and inter-assay coefficients of variation were below 15% for all the assays.

General blood tests were performed by our hospital laboratory; the red blood cell and haemoglobin levels were measured using the IDEXX VetAutoread™ Hematology Analyzer (Thermo Fisher Scientific, Waltham, MA, USA).

### 2.3. Statistical Analysis

A Shapiro–Wilk test showed that the data were not normally distributed.

A Kruskal–Wallis test was used to compare the groups. A Bonferroni’ comparisons test was performed following the Kruskal–Wallis test, in case of any significant differences. Receiver operating characteristic (ROC) curves were not performed due to the relatively small number of patients that were involved. The correlations between the CHI3L1 levels, the motor and cognitive symptoms, and the laboratory and instrumental parameters were calculated by the Spearman rank correlation (rs). The level of significance for all the statistical tests was set at 0.05.

We used the program SPSS Statistic V26 (Chicago, IL, USA) to perform the statistical calculations.

All the patients signed a written informed consent form. The study design was approved by the Ethical Committees of the Turin ALS Center (Comitato Etico Azienda Ospedaliero-Universitaria Città della Salute e della Scienza, Torino) (n° 0011613, 3 February 2020).

## 3. Results

We performed a pilot cross-sectional study, in which we enrolled 19 HCs, 44 MND patients, 7 hereditary spastic paraplegia (HSP) patients, and 9 MND mimics (including those with myelopathy, radiculopathy, axonal neuropathies, and post-poliomyelitis syndrome), seen at the ALS Center in Turin, Molinette Hospital between January 2019 and August 2022. Our 44 MND patients included 25 with ALS, 12 with ALS with cognitive impairment (ALS/FTD or ALSci, using Strong classification [20]), 5 with PLS, and 2 with PMA.

The participant demographic and clinical characteristics are summarized in Table 1.

None of the patients or the controls included in the study suffered from active cancer, infectious diseases, autoimmune diseases, diabetes, liver diseases, or had ongoing immunosuppressive therapies.

### 3.1. Plasmatic CHI3L1 in Differential Diagnosis

**Figure 1:**
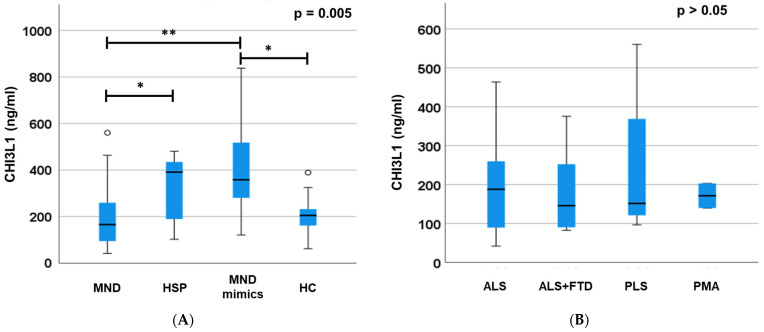
CHI3L1 plasmatic levels in MND, MND mimic, HSP, and HC. In MND mimics, we highlighted an important increase in CHI3L1 plasmatic levels compared to MND and HC (**A**). Moreover, we observed a slight difference between the MND and the HSP group. We also tried to search for differences in levels of CHI3L1 among the MND sub-groups (PLS, ALS/FTD, ALS, and PMA), but unsuccessfully (**B**). Error bars (whiskers) showed 95% confident intervals. * *p* < 0.05; and ** *p* < 0.005. MND: motor neuron diseases; HSP: hereditary spastic paraplegia, HC: healthy controls; ALS: amyotrophic lateral sclerosis; FTD: frontotemporal dementia; PLS: primary lateral sclerosis; and PMA: progressive muscular atrophy.

The CHI3L1 plasma levels were different between the groups (*p* = 0.005; Figure 1A). Significantly, the Bonferroni’s correction showed that the MND mimics had significantly higher levels of CHI3L1 compared with the MND patients (*p* = 0.002) and HCs (*p* = 0.023), and that the HSP patients had higher levels of CHI3L1 compared with the MND patients (*p* = 0.03). No difference was observed between the HSP and MND patients versus the HCs (*p* > 0.05). Introducing sex and age as covariates, we confirmed the differences in the CHI3L1 levels between the MND mimics and both the MND patients and HCs (*p* = 0.004). A sub-group analysis of the MND patients (divided into PLS, ALS, ALS + FTD, and PMA, Figure 1B) did not show any difference in the CHI3L1 levels (*p* > 0.05). No differences were noticed in the CHI3L1 levels between the genetic and non-genetic forms of ALS, nor between fast- and slow-progression ALS (*p* > 0.05).

### 3.2. Correlation of Plasmatic CHI3L1 with Motor and Cognitive Symptoms and with Laboratory and Instrumental Parameters

**Figure 2:**
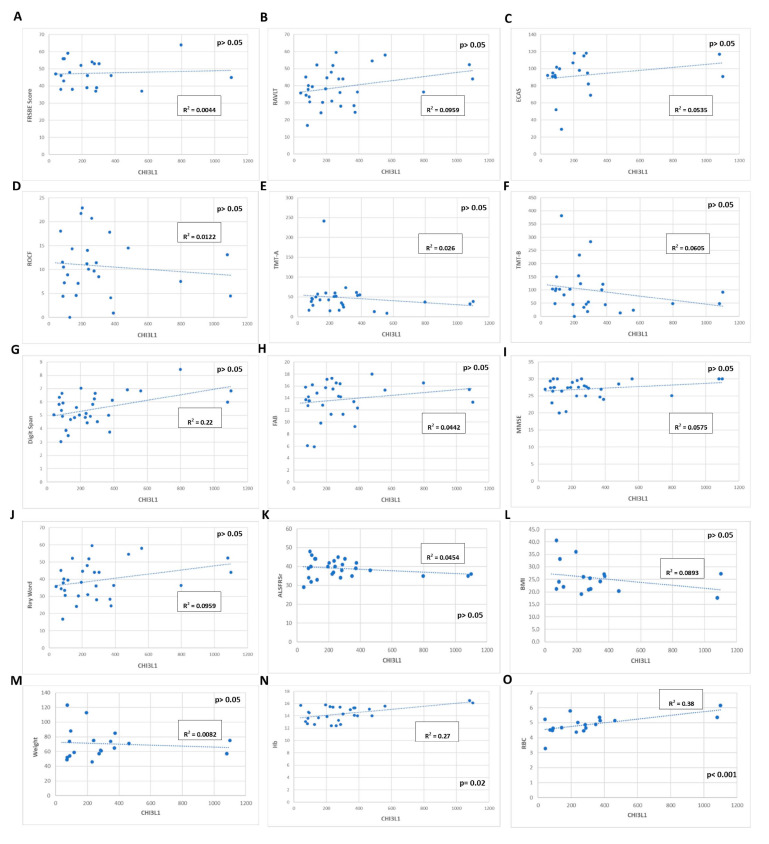
Correlation between CHI3L1 plasmatic levels and clinical, neuropsychological, and laboratory data in MND patients. We did not observe any statistical correlation (*p* > 0,05) between CHI3L1 plasmatic levels and any neuropsychological test (**A**–**J**), ALSFRSr scale (**K**), weight, or BMI (**L**,**M**). The only correlation we identified is between CHI3L1 plasmatic levels and haemoglobin (**N**), and CHI3L1 plasmatic levels and red blood cells (**O**). ALSFRSr: ALS Functional Rating Scale revised; Hb: haemoglobin; RBC: red blood cells; FRSBE: Frontal Systems Behavior Scale; RAVLT: Rey Auditory Verbal Learning Test; ECAS: Edinburgh Cognitive and Behavioural ALS Screen; ROCF: Rey–Osterrieth complex figure; TMT-A: Trail Making Test A; TMT-B: Trail Making Test B; FAB: Frontal Assessment Battery; MMSE: mini–mental state examination; BMI: body mass index; R: Pearson coefficient.

CHI3L1 does not correlate with weight, BMI, ALSFRSr, the MRC total score and sub-scores, forced vital capacity (FVC), forced expiratory volume in the 1st second (FEV1), the PR of the disease at the time of blood sampling, and blood examination, except for with red blood cells (RBC) and haemoglobin (respectively, *p* < 0.001, r = 0.63 and *p* = 0.022, r = 0.52, Figure 2N,O). Since the levels of RBC and haemoglobin are influenced by the BMI, we performed a multiple linear regression that confirmed the significance of the correlation of CHI3L1 with RBC and haemoglobin (respectively, *p* < 0.0001, r = 0.96 and *p* = 0.016, r = 0.73). None of the neuropsychological tests correlated with the CHI3L1 plasma levels (*p* > 0.05).

## 4. Discussion

This preliminary cross-sectional study highlights the possible benefits of dosing the CHI3L1 plasma levels in patients with suspected MNDs to differentiate them from MND mimics. In fact, the CHI3L1 plasma levels were increased in the evaluated MND mimics (including acute myelopathy, radiculopathies, and neuropathies) compared with the MND patients and HCs. These data are consistent with the known increase in CHI3L1 in diseases in which inflammation plays a crucial role, such as cancers or autoimmune/dysimmune diseases [12,21]. Therefore, the increase in CHI3L1 in the MND mimics group could be explained by the presence of an active peripheral inflammatory component.

Moreover, the CHI3L1 levels were statistically significantly higher in the HSP patients compared with the MND patients, likely due to the high frequency of neuropathies in HSP, in which there is usually an inflammatory component.

Contrarily to other studies performed on CSF, in which CHI3L1 allowed for differentiation between MND patients and HCs [6,7], the plasmatic levels of CHI3L1 do not permit the identification of these two groups. This could be due to the low ability of CHI3L1 to pass through the blood–brain barrier, and to the plasmatic release of CHI3L1 from other tissues, which masks its increase from the central nervous system.

In the MNDs cohort, we also correlated the CHI3L1 levels with several clinical, laboratory, and neuropsychological parameters that were collected within one month of the plasma collection, but we did not observe any association. In particular, in contrast with previous evidence from CSF [7], the plasmatic levels of CHI3L1 did not correlate with the score of the executive and visuo–spatial neuropsychological tests. The only correlation that was observed was with haemoglobin and RBC; this is consistent with previous evidence, since CHI3L1 seems to be more concentrated in RBCs [22].

This study has some limitations. First, the number of patients included was relatively small, in particular in the MND mimics group. Second, we have not included all the types of MND mimics that should be considered in a more exhaustive future study (i.e., myasthenia gravis, syringomyelia, adult polyglucosan body disease, Kennedy’s disease, and inclusion body myositis). Moreover, we have not performed a comparison of the plasma levels of the CHI3L1 with the CSF ones, and we have not included other fluid biomarkers, since it was a pilot study.

To validate our data and observe the variation of CHI3L1 and other biomarkers throughout the disease course, further longitudinal and multicentre studies with a higher number of patients both in the MND and MND mimics groups are needed. Finally, a comparison between the plasmatic and CSF levels of the CHI3L1 protein is mandatory, in order to confirm that the plasmatic levels of CHI3L1 do not satisfactorily reflect the CSF ones.

## 5. Conclusions

The measurement of the plasmatic levels of CHI3L1 could be useful in the differential diagnosis between MNDs and MND mimics. This is an important issue, since the early diagnosis of an MND is a determinant in the early starting of neuroprotective therapy and in clinical trial recruitment.

Further multicentre and longitudinal studies on a larger patient cohort, testing alongside other fluid biomarkers, are needed to better explain the role of CHI3L1 in the diagnosis and prognosis of MNDs and, also, of MND mimics.

## Figures and Tables

**Table 1:** Participant demographic and clinical information at blood sampling.

Patients Group	n°	Sex M/F	Age ± SD (Year)	Genetic Mutation	ALSFRSr ± SD	FVC ± SD
**MND**	44	20/24	63 ± 13	7/44	39.5 ± 4.8	98 ± 19
**MND mimics**	9	5/4	62 ± 15	N/A	N/A	N/A
**HSP**	7	5/2	64 ± 8	7/7	N/A	N/A
**HC**	19	10/9	60 ± 9	N/A	N/A	N/A

N/A: not assessed; SD: Standard Deviation; ALSFRSr: ALS Functional Rating Scale revised; FVC: Forced Vital Capacity.

## Data Availability

The data presented in this study are available on request from the corresponding author.
